# Degradation of Muscle Quality in Hybrid Grouper (♀ *Epinephelus fuscoguttatus* × ♂ *Epinephelus lanceolatu*) Due to Oxidative Damage Caused by Ingestion of Oxidized Fish Oil

**DOI:** 10.3389/fnut.2022.840535

**Published:** 2022-02-15

**Authors:** Xiaobo Yan, Zhihao Li, Xiaohui Dong, Beiping Tan, Simiao Pan, Tao Li, Shuisheng Long, Weibin Huang, Xiangxiang Suo, Yuanzhi Yang

**Affiliations:** ^1^Laboratory of Aquatic Nutrition and Feed, College of Fisheries, Guangdong Ocean University, Zhanjiang, China; ^2^Aquatic Animals Precision Nutrition and High Efficiency Feed Engineering Research Center of Guangdong Province, Zhanjiang, China; ^3^Key Laboratory of Aquatic, Livestock and Poultry Feed Science and Technology in South China, Ministry of Agriculture, Zhanjiang, China

**Keywords:** fresh fish oil, oxidized fish oil, muscle quality, flavor, grouper, oxidative damage

## Abstract

The aim of the study was to investigate the effects of fresh fish oil (FFO) and oxidized fish oil (OFO) diets on the muscle quality of hybrid grouper (♀ *Epinephelus fuscoguttatus* × ♂ *E. lanceolatu*). Hybrid grouper were fed with diets containing 9% FFO or OFO for 60 days. Muscle sample were collected at 0, 30, and 60 days and the selected indexes of muscle were measured. Malondialdehyde (MDA), reactive oxygen species (ROS), total cholesterol (TC) and triglycerides (TG) in grouper muscle accumulated gradually with prolonged ingestion time, especially OFO group. Total saturated fatty acids (ΣSAFA) was significantly reduced and total polyunsaturated fatty acids (ΣPUFA) was significantly increased of muscle in FFO group; meanwhile, the muscle ΣSAFA and monounsaturated fatty acids (ΣMUFA) contents in the OFO group were significantly higher than those in the FFO group and the ΣPUFA (especially C22:5n3, C22:6n3) contents was significantly lower than that in the FFO group at 60 days. Consumption of OFO diet for 60 days reduced the diversity of volatile compounds, significantly reduced the content of total esters and increased the content of total aldehydes and total aromatics in grouper muscle. Furthermore, ingestion of OFO diet significantly reduced the mRNA expression of fraction growth factors and antioxidant genes in the muscle of grouper. In conclusion, the increasing MDA content in FO and the oxidative rancidity of PUFA can cause the deterioration of grouper quality and flavor due to oxidative muscle damage.

## Introduction

Lipid is an essential nutrient for aquaculture animals and is an important component of tissue cells, it has the function of providing essential fatty acids, storing and supplying energy and saving protein. Fish oil (FO) is widely used in the aquafeed industry because of its high unsaturated fatty acids (HUFAs) content, especially eicosapentaenoic acid (EPA) and docosahexaenoic acid (DHA), which are essential fatty acids for mariculture animals. However, HUFAs are susceptible to oxygen, moisture, light and the metal in the feed additives and other factors, very easy to oxidation and decomposition into alcohols, ketones, aldehydes, and other harmful substances in the production, use and storage process (1). Ultimately, it has a serious impact on the health and quality of the animal. The lipid content in the feed of mariculture fish is generally 12–15% (2), and the farming objects are generally concentrated in subtropical or tropical areas such as high temperature and high humidity, which provides suitable environmental conditions for the oxidation of FO in feed. Therefore, the problem of FO oxidation in marine fish feed has seriously affected the sustainable development of the mariculture industry.

With the development of the world economy and the improvement of living standards, human beings are also demanding higher quality seafood, and the problem of deterioration of fish health and quality caused by feed rancidity has become one of the important problems that cannot be ignored in the aquaculture industry today. In recent years, there has been an increasing amount of research on oxidized lipids in feed related to oxidative stress. Huang and Huang (3) showed that ingestion of oxidized fish oil (OFO) feed reduced growth performance and increased malondialdehyde (MDA) content of liver in juvenile hybrid tilapia (*Oreochromis niloticus* × *O. aureus*); Zhang et al. (4) showed that OFO feed caused liver lipid deposition in loach (*Misgurnus anguillicaudatus*). These studies focused on the effects of OFO on growth performance, liver biochemical parameters, and antioxidant capacity (5). In addition, oxidation of FO may not only reduce the nutritional value of the feed itself, but may also adversely affect fish muscle quality (amino acid composition, fatty acid composition, vitamin E content, flavor, shelf life, etc.) (6). Previous studies have also shown that ingestion of OFO feeds causes increased MDA and decreased *n*−3 polyunsaturated fatty acids (PUFAs) content in the muscles of fish such Japanese sea bass (*Lateolabrax japonicus*) (7), Japanese flounder (*Paralichthys olivaceus*) (8), largemouth bass (*Micropterus salmoides*) (5), Atlantic cod (*Gadus morhua*) (9), which seriously affects fish muscle quality and nutritional value. However, there is a lack of systematic studies on the effect of OFO on the nutritional value and quality of fish muscle.

Hybrid grouper (♀ *Epinephelus fuscoguttatus* × ♂ *E. lanceolatu*) has become one of the most important breeding species along the southeast coast of China due to its fast growth rate, tasty meat, high nutritional value, strong disease resistance, and adaptability to the environment (10). The annual production of grouper can reach about 200,000 tons in China, which is a high-quality source of protein. At present, research on pearl grouper is mainly focused on nutritional requirements and immunity (11), there are few reports about its muscle quality. Muscle is the primary part of human consumption, and its nutritional composition, flavor substance composition and quality are related to human health and consumption preferences. In the era of intensive development of aquaculture, preventing oxidation of FO in feed becomes challenging, and it remains to be explored whether the ingestion of OFO feed will have a negative impact on the muscle quality and nutritional value of grouper. Therefore, the study was conducted to compare the muscle nutrient composition, fatty acid composition, amino acid composition, flavor substance composition and flesh quality-related genes expression by feeding fresh fish oil (FFO) diet and OFO diet in different rearing periods using hybrid grouper as the research object. The results can provide basic data for regulating the decline of muscle quality caused by the consumption of OFO diet.

## Materials and Methods

### Experimental Diets

Two iso-lipid (10.50%) and iso-protein (48.92%) diets were formulated containing 9% FFO and OFO, respectively. The peroxide value (POV) of the OFO was 122.85 ± 1.76 mmol/kg, and the malondialdehyde (MDA) content was 121 mg/kg. All ingredients were crushed and sieved through a 60 mesh sieve, then thoroughly mixed using the progressive enlargement method after accurate weighing. Then added oil and lecithin, rubbed them manually and putted them into V-type vertical mixer after sieving and mixed them evenly, and then added distilled water (30~40%) to mix them evenly. The diets were processed into 2.5 mm diameter pellets by a twin-screw extruder (F−26, South China University of Technology, Guangdong Province, China), air-dried to about 10% moisture content at room temperature, then ground and sieved to an appropriate size and stored in ziploc bags at −20°C until use. The ingredients and approximate compositions of the diets were shown in Table 1.

**Table 1:** Composition and nutrients levels of the test diets (air dry matter %).

**Ingredients**	**FFO**	**OFO**
Fish meal	40.00	40.00
Wheat gluten	14.00	14.00
Corn gluten meal	8.00	8.00
Soybean meal	9.00	9.00
Wheat flour	14.35	14.35
Fresh fish oil	9.00	0.00
Oxidized fish oil^a^	0.00	9.00
α-starch	3.00	3.00
Calcium monophosphate	1.00	1.00
Choline chloride	0.50	0.50
Compound additives^b^	1.00	1.00
Attractant	0.15	0.15
Total	100.00	100.00
Proximate composition^c^		
Crude protein	49.47	48.96
Crude lipid	10.42	10.53

*^a^Oxidized fish oil is made from fresh fish oil by heating and aeration. Its POV is 122.85 ± 1.76 mmol/kg and MDA content is 121 mg/kg. There are significant differences of POV and MDA between oxidized fish oil and fresh fish oil.*

*^b^Compound additives contenting a variety of vitamins and minerals were obtained from Beijing Enhalor Biotechnology Co., Ltd.*

*^c^Measured value*.

### Fish and Feeding Trial

This study was approved by the ethics review board of the Institutional Animal Care and Use Committee at Guangdong Ocean University in Zhanjiang, China. Hybrid groupers (♀ *Epinephelus fuscoguttatus* × ♂ *E. lanceolatu*) were purchased from a local farm at Donghai Island (Zhanjiang, China) and were temporarily raised in an outdoor cement pond (5 m × 4 m × 1.8 m) to the required specification for the experiment. After fasting for 24 h, 180 fish (21.36 ± 0.03 g) were randomly distributed into six tanks (500 L). FFO and OFO diets were fed daily at 8:00 and 16:00, respectively, until apparent satiation was observed and trial last 60 days. About 70% of the water was exchanged to maintain water quality every day. Every tank was provided with one piece of polyvinylchloride (PVC) pipe of 20.0 cm (diameter) ×30.0 cm (length) as shelter for the fish. Natural illumination, water temperature was maintained at 29 ± 1°C, salinity was 28, pH was 7.9 ± 0.1, and the dissolved oxygen concentration was kept at above 5.0 mg/L. The concentrations of total ammonia and nitrite remained below 0.05 mg/L.

### Sample Collection

Sample collection of the experiment was carried out at 0 day (initial comparison), 30, and 60 days and the fish were starved for 24 h before each sampling. This allows monitoring a dynamic process of change in muscle quality of grouper after ingestion of diet. After anesthetized with eugenol (1:10,000), stripping the skin of the fish with bone forceps and then separating the back muscles. The samples used to determine proximate compositions, amino acids, and fatty acids content are kept on ice. The samples for enzyme activity and volatile substances measured are placed in 2.0 ml lyophilization tubes and stored in liquid nitrogen. All the above samples were measured immediately after collection. Some samples were placed in tubes with RNA later overnight at 4°C and then transferred to −80°C refrigerator until mRNA expression assay.

### The Methods of Analysis

The peroxide value (GB 5009.227-2016) and MDA (GB/T 28717-2012) content of OFO were determined by Chinese standard methods. Proximate analysis of the diets and muscle followed the methods specified by AOAC (12). Moisture was determined by drying at 105°C, crude protein was determined by multiplying nitrogen by 6.25 (Kjeltec^TM^ 8400, Denmark), crude lipid was determined by Soxhlet extraction (using petroleum ether as solvent).

Fatty acid methyl esters were prepared by acid-catalyzed transmethylation of total lipids using boron trifluoride-methanol and were analyzed by gas chromatograph (7890A, Agilent Technologies Inc., US). Amino acid determination using Chinese standard methods (GB/T18246-2019). Muscle total protein (TP), MDA, reactive oxygen species (ROS), total cholesterol (TC), and triglycerides (TG) were analyzed using commercial ELISA kits (Shanghai Enzyme-linked Biotechnology Co., Ltd., Shanghai, China). The volatile substances were determined by headspace solid-phase microextraction (HS-SPME) and gas chromatography-mass spectrometry coupled (Agilent 7890A-5975C). The data processing was based on the computer search of the NIST08 spectral library, supplemented by manual analysis of the spectra and qualitative analysis combined with existing literature reports. The relative percentages of various volatile components were obtained by the peak area normalization method through the excel data processing system.

RNA extraction and cDNA synthesis were performed by TransGen Biotech (Beijing, China) RNA kit and Accurate Biology Evo M-MLV kit (Hunan, China), respectively. Real-time quantitative polymerase chain reaction (RT-qPCR) was performed in a 384-well-plate with a 10 μL reaction volume containing 5 μL of SYBR® Green Real-time PCR Master Mix, 0.8 μL of each primer, 1 μL of cDNA sample, and 3.2 μL of RNse Free dH_2_O. Primers of the reference gene (β-actin) and target genes were designed according to published sequences of groupers (Table 2). The relative expression levels of selected genes were calculated using the 2^−ΔΔCt^ method.

**Table 2:** Primers sequences used for RT-qPCR.

**Primers names**	**Forward and reverse primers sequence (5′-3′)**	**Genbank accession No**.
*β-Actin*-F/R	GGCTACTCCTTCACCACCACA/TCTGGGCAACGGAACCTCT	AY510710.2
*MyoG*-F/R	TTATCCCGTGGTCCAGAGGT/GGTGTCGGGTTCATGCAGTA	XM_033625713.1
*MyoD*-F/R	CTGAAAGTGTGGAGGCTCGT/GATGAACACTGTGCGAAGCG	XM_033635008.1
*Myf5*-F/R	CCGTCCCAGGTCTACTACGA/GTACCCTCACATGCTCGTCC	HM190249.1
*MRF4*-F/R	GAGAGAGGGGCGCAACAATA/ACGGAACATTATCCTGGCCC	HM190248.1
*MSTN2*-F/R	ATTGTTTCCCGGGTGCTCAT/GTTGAAGGTCGCCTCCAGAA	KR269829.1
*IGF1*-F/R	AGATGTACTGTGCACCTGCC/GTCCTACGCTCTGTGCCTTT	AY776159.1
*IGF2*-F/R	GTTGTGGAAATAGCGTCGGC/TCCTCTACGATCCCACGGTT	AY776158.1
*COL1A1*-F/R	CACTGAGGCATCCCAGAACC/TGTGTGACGTGCATCCATCT	JN112557.1
*COL1A2*-F/R	GGAAGCACAGCCATATCCTGA/GGCATGGACAGTGTCAACAGA	XM_033636413.1
*Hsp70*-F/R	CTTGCAAGAAGTGGCCAACA/AAAGCCATCTTCCTGCCTTGT	EU816600.1
*Hsp90*-F/R	AACGACAAGGCTGTGAAGGAC/TTCTGTAGATGCGGTTGGAGTG	HQ441094.1
*SOD*-F/R	TGGAAACACCTTTCCCCCAC/CTGACAGGGTAAAGCATGGC	AY735008.1
*CAT*-F/R	CGCGGGAAGCAAAGATTCAG/CCGCAGTTTCCAGTGTGTTG	KT884509.1
*GPX*-F/R	TCCTCTGTGGAAGTGGCTGA/TCATCCAGGGGTCCGTATCT	HQ441085.1

*MyoG, myogenin; MyoD, myogenic differentiation; Myf5, myofactar5; MRF4, myogenic regulatory factor 4; MSTN2, myostatin type 2; IGF1, insulin-like growth factor 1; IGF2, insulin-like growth factor 2; COL1A1, collagen type I alpha1; COL1A2, collagen type I alpha2; Hsp70, heat shock protein 70; Hsp90, heat shock protein 90; SOD, copper/zinc superoxide dismutase; CAT, catalase; GPX, glutathione peroxidase*.

### Statistical Analysis

All data were firstly examined for homogeneity of variance using SPSS version 20.0 (SPSS Inc., USA). The results of different time periods were subjected to one-way analysis of variance followed by Tukey of tests significant differences among treatment groups, and probability values of *P* < 0.05 were deemed to be statistically significant. Samples from different treatments at the same time were analyzed using independent sample *T*-tests. The results are presented as means ± standard error (SEM).

## Results

### Muscle Composition and Biochemical Indexes

As shown in Table 3, there were no significant effect of FO quality in feed on muscle moisture content, crude lipid, and crude protein of grouper. Meanwhile, muscle crude protein, and crude lipid were not affected by feeding time (*P* > 0.05). The muscle moisture of grouper feeding on both FFO and OFO diets decreased significantly with the increase of rearing time (*P* < 0.05). Duration of ingestion of FFO feed did not affect TP, MDA, ROS, and TC content of grouper muscle (*P* > 0.05). However, TG content increased gradually with the prolongation of feeding time in OFO group and muscle TG content was significantly higher at 60 days than the initial value (*P* < 0.05). MDA, TC, and TG contents of grouper muscle increased with the extend of feeding time of OFO feed, and the MDA and TG contents were significantly higher than the initial values at 30 d, and the TC contents were significantly higher at 60 days (*P* < 0.05). At 30 days, intake of OFO significantly increased the MDA content of grouper muscle (*P* < 0.05), and ROS, TC, and TG contents were also higher than those in the FFO group, but there was no significant difference (*P* > 0.05). At 60 days, the muscle TP content in the OFO group was significantly lower than that in the FFO group (*P* < 0.05), but there were no significant differences of other indicators between two groups (*P* > 0.05).

**Table 3:** Muscle composition and biochemical index of grouper fed different diets.

**Items**	**0 day**	**30 FFO**	**30 OFO**	**60 FFO**	**60 OFO**
Moisture %	78.42, 0.15^Bb^	71.81, 0.15^A^	72.01, 0.50^a^	71.92, 0.44^A^	72.70, 0.07^a^
Crude lipid %	4.90, 0.20	4.98, 0.49	4.82, 0.31	5.54, 0.64	6.31, 1.08
Crude protein %	81.72, 1.29	83.67, 1.09	82.44, 0.16	80.58, 0.94	82.26, 0.34
TP mg/ml	0.60, 0.02	0.60, 0.03	0.58, 0.03	0.60, 0.02**	0.55, 0.03**
MDA nmol/mg.pro	17.62, 0.18^a^	18.51, 0.16*	22.76, 1.01^b*^	18.31, 0.43	19.52, 0.84^ab^
ROS U/mg.pro	378.89, 16.28	461.86, 56.62	480.74, 44.00	505.32, 31.62	464.15, 19.87
TC μmol/mg.pro	14.40, 0.16^a^	16.33, 0.97	17.70, 0.72^ab^	16.31, 0.44	18.52, 0.84^b^
TG μmol/mg.pro	12.33, 0.03^Aa^	13.64, 0.38^AB^	17.89, 1.48^b^	15.81, 0.52^B^	15.92, 0.88^ab^

The results are presented as the means ± SEM (n = 3). Different superscript capital letters in the same row indicate significant differences in the FFO group at different time point, and different lowercase letters indicate significant differences in the OFO group at different time point. ^*^Indicates significant differences of two treatments at 30 days, ^**^indicates significant differences of two treatments at 60 days (P < 0.05). TP, total protein; MDA, malondialdehyde; ROS, reactive oxygen species; TC, total cholesterol; TG, triglycerides.

### Muscle Amino Acids

Results displayed in Table 4. For two diets, feeding time had no influence on the tyrosine, lysine, valine, leucine, isoleucine, histidine, arginine, threonine, and alanine relative content of grouper muscle (*P* > 0.05). Significant decrease of muscle methionine, phenylalanine, total indispensable amino acid, aspartic acid, serine, glutamic acid, total dispensable amino acid, and total amino acids content in FFO group at 30 days compared to initial values (*P* < 0.05), and at 60 days they reverted to no significant difference from the initial value. Nevertheless, the accumulation of proline increased significantly with the ingestion time (*P* < 0.05). Aspartic acid and total amino acids contents significant decrease when ingestion OFO feed for 30 days (*P* < 0.05), and reverted to no significant difference from the initial value at 60 days. As with the FFO diet, the proline level in the muscle of grouper feeding on OFO also accumulated significantly with the feeding time (*P* < 0.05). Ingestion of OFO feeds barely affected muscle amino acids in grouper except for phenylalanine compared with FFO group (*P* > 0.05). After 60 days of cultivation, the phenylalanine content in the muscle of the OFO group was significantly higher than that of the FFO group (*P* < 0.05).

**Table 4:** Muscle amino acids content of grouper fed different diets.

**Amino acids**	**0 day**	**30 FFO**	**30 OFO**	**60 FFO**	**60 OFO**
Tyrosine	2.71, 0.00	2.28, 0.17	2.70, 0.09	2.65, 0.01	2.75, 0.04
Lysine	7.36, 0.00	6.87, 0.21	7.25, 0.12	7.24, 0.05	7.51, 0.13
Valine	3.22, 0.00	3.10, 0.09	3.28, 0.04	3.14, 0.07	3.30, 0.08
Methionine	2.40, 0.00^B^	2.20, 0.07^A^	2.35, 0.03	2.35, 0.02^AB^	2.41, 0.04
Leucine	6.23, 0.00	5.84, 0.19	6.12, 0.07	6.13, 0.03	6.32, 0.11
Isoleucine	3.20, 0.00	2.93, 0.12	3.17, 0.05	3.05, 0.07	3.25, 0.09
Phenylalanine	3.19, 0.00^Bab^	2.78, 01.5^A^	3.16, 0.09^a^	3.13, 0.01^AB**^	3.39, 0.06^b**^
Histidine	1.85, 0.00	1.78, 0.06	1.88, 0.03	1.89, 0.02	1.89, 0.03
Arginine	4.65, 0.00	4.63, 0.05	4.64, 0.13	4.68, 0.03	4.86, 0.08
Threonine	3.68, 0.00	3.52, 0.08	3.64, 0.06	3.63, 0.08	3.76, 0.06
Indispensable amino acid (IAA)	38.49, 0.00^B^	35.93, 0.96^A^	38.19, 0.54	37.89, 0.30^AB^	39.45, 0.70
Alanine	4.93, 0.00	4.94, 0.03	4.96, 0.04	4.93, 0.06	5.10, 0.08
Aspartic acid	9.26, 0.00^Bb^	8.13, 0.18^A^	8.47, 0.13^a^	9.08, 0.05^B^	8.67, 0.16^a^
Serine	3.35, 0.00^B^	3.18, 0.09^A^	3.27, 0.04	3.32, 0.06^AB^	3.33, 0.02
Glutamic acid	12.67, 0.00^B^	11.79, 0.29^A^	12.43, 0.24	12.48, 0.11^AB^	12.73, 0.17
Glycine	4.12, 0.00^a^	4.60, 0.25	4.46, 0.11^b^	4.36, 0.10	4.39, 0.10^ab^
Proline	3.11, 0.00^Aa^	3.62, 0.08^B^	3.63, 0.11^b^	3.93, 0.08^C^	3.92, 0.02^c^
Dispensable amino acid (DAA)	37.44, 0.00^B^	36.26, 0.23^A^	37.21, 0.46	38.10, 0.39^B^	38.14, 0.51
Total amino acids (TAA)	75.93, 0.00^Bb^	72.19, 1.19^A^	72.19, 1.19^a^	76.00, 0.62^B^	75.99, 0.62^b^
IAA/TAA	0.51, 0.00	0.50, 0.01	0.51, 0.00	0.50, 0.00	0.51, 0.00

The results are presented as the means ± SEM (n = 3). Different superscript capital letters in the same row indicate significant differences in the FFO group at different time point, and different lowercase letters indicate significant differences in the OFO group at different time point. ^*^Indicates significant differences of two treatments at 30 days, ^**^indicates significant differences of two treatments at 60 days (P < 0.05).

### Muscle Fatty Acids

Muscle C15:0, C17:0, C18:0, and total saturated fatty acids (∑SAFA) decreased significantly in FFO group with the extended ingestion time (*P* < 0.05), while there was no significant difference of SAFA in OFO group (*P* > 0.05) (Table 5). While no significant variation in C14:0 and C16:0 was observed after 30 days of FFO ingestion (*P* > 0.05), a significant decrease was observed after 60 days (*P* < 0.05). Although, there was no significant difference of SAFA between FFO and OFO groups at 30 days (*P* > 0.05), C16:0 and ∑SAFA were significantly higher in OFO group than those in FFO group at 60 days (*P* < 0.05). The muscle C20:1n9, C22:1n9, and C24:1n9 content of grouper muscle was significantly increased after the ingestion of FFO diets (*P* < 0.05), although there was no significant difference about C15:1n7 and C18:1n9 in FFO group at 30 days compare with the initial value (*P* > 0.05), but they decreased significantly at 60 days (*P* < 0.05). C16:1n7 and monounsaturated fatty acids (∑MUFA) decreased significantly after 30 days of ingestion of FFO feed (*P* < 0.05), but then recovered to non-significant differences from the initial values at 60 days (*P* > 0.05). Furthermore, ingestion of OFO diet for 60 days significantly increased muscle C18:1n9 and ∑MUFA contents compared to ingestion of FFO diet (*P* < 0.05). The muscle C20:3n6, C20:4n6, C20:3n3, C20:5n3, C22:2n3, C22:6n3, total polyunsaturated fatty acids (∑PUFA) contents of FFO group increased gradually with the prolonged ingestion time, all of which were significantly higher than the initial values at 60 days (*P* < 0.05). In contrast, C18:2n6, C18:3n3, and C20:2n6 contents were significantly reduced after ingestion of FFO (*P* < 0.05). Similarly, C18:2n6 and C18:3n3 in muscle of grouper feeding on OFO diet also decreased significantly with feeding time (*P* < 0.05). Compared with FFO group, OFO diet significantly reduced the C20:5n3, C22:6n3, and ∑PUFA content after 60 days (*P* < 0.05).

**Table 5:** Muscle fatty acids of grouper fed different diets %.

**Fatty acids**	**0 day**	**30 FFO**	**30 OFO**	**60 FFO**	**60 OFO**
C14:0	2.83, 0.00^B^	2.86, 0.06^B^	3.08, 0.50	2.03, 0.11^A^	2.51, 0.45
C15:0	0.44, 0.00^C^	0.37, 0.02^B^	0.43, 0.07	0.26, 0.01^A^	0.38, 0.08
C16:0	22.35, 0.00^B^	21.97, 0.22^B^	22.24, 2.47	17.71, 0.37^A**^	21.72, 3.25**
C17:0	0.9, 0.00^C^	0.78, 0.01^B^	0.87, 0.06	0.68, 0.01^A^	0.81, 0.07
C18:0	8.64, 0.00^C^	7.49, 0.15^B^	7.73, 0.39	6.61, 0.13^A^	7.95, 0.64
C20:0	0.48, 0.00	0.54, 0.03	0.56, 0.05	0.47, 0.02	0.59, 0.08
C21:0	0.09, 0.00	0.07, 0.07	0.15, 0.01	0.14, 0.00	0.04, 0.04
C22:0	0.25, 0.00^AB^	0.27, 0.01^B^	0.28, 0.03	0.24, 0.01^A^	0.28, 0.04
C24:0	0.19, 0.00	0.00, 0.00	0.00, 0.00	0.00, 0.00	0.00, 0.00
∑SAFA	36.16, 0.00^C^	34.34, 0.57^B^	35.34, 3.53	28.15, 0.39^A**^	34.28, 4.54**
C15:1n7	0.2, 0.00^B^	0.17, 0.01^AB^	0.18, 0.04	0.15, 0.01^A^	0.06, 0.06
C16:1n7	3.46, 0.00^A^	4.03, 0.06^B^	4.28, 0.51	3.37, 0.16^A^	3.93, 0.53
C18:1n9	21.81, 0.00^B^	21.55, 0.42^B^	22.24, 1.97	18.99, 0.25^A**^	23.45, 2.83**
C20:1n9	1.87, 0.00^Aa^	2.80, 0.05^C^	2.84, 0.26^ab^	2.65, 0.02^B^	3.34, 0.51^b^
C22:1n9	0.31, 0.00^A^	0.54, 0.03^B^	0.58, 0.10	0.52, 0.01^B^	0.70, 0.14
C24:1n9	0.56, 0.00^Aa^	0.91, 0.03^B^	0.93, 0.08^ab^	0.86, 0.01^B^	1.09, 0.17^b^
∑MUFA	28.2, 0.00^A^	29.99, 0.59^B^	31.07, 2.89	26.53, 0.41^A**^	32.57, 4.11**
C18:2n6	18.56, 0.00^Bb^	10.18, 0.11^A^	11.22, 0.39^a^	10.34, 0.32^A^	10.65, 1.04^a^
C18:3n6	0.08, 0.00^B^	0.00, 0.00^A^	0.09, 0.05	0.14, 0.00^C^	0.05, 0.05
C18:3n3	1.39, 0.00^Bb^	0.89, 0.05^A^	0.95, 0.12^ab^	0.94, 0.01^A^	0.76, 0.17^a^
C20:2n6	0.55, 0.00^C^	0.45, 0.01^A^	0.46, 0.02	0.50, 0.01^B^	0.51, 0.04
C20:3n6	0.18, 0.00^A^	0.22, 0.01^B^	0.20, 0.03	0.26, 0.01^C^	0.17, 0.09
C20:4n6	1.04, 0.00^A^	1.06, 0.02^A^	0.99, 0.21	1.29, 0.02^B^	0.97, 0.28
C20:3n3	0.12, 0.00	0.07, 0.07	0.10, 0.05	0.16, 0.00	0.06, 0.06
C20:5n3	5.2, 0.00^A^	11.33, 0.44^B^	9.53, 2.60	15.74, 0.30^C**^	10.04, 3.66**
C22:2n3	0.07, 0.00	0.00, 0.00	0.00, 0.00	0.03, 0.03	0.00, 0.00
C22:6n3	8.45, 0.00^A^	11.49, 0.60^B^	10.07, 3.05	15.87, 0.32^C**^	9.96, 3.94**
∑PUFA	35.64, 0.00^A^	35.68, 1.17^A^	33.59, 6.42	45.29, 0.82^B**^	33.15, 8.64**

The results are presented as the means ± SEM (n = 3). Different superscript capital letters in the same row indicate significant differences in the FFO group at different time point, and different lowercase letters indicate significant differences in the OFO group at different time point. ^*^Indicates significant differences of two treatments at 30 days, ^**^indicates significant differences of two treatments at 60 days (P < 0.05).

### Muscle Volatile Compounds

**Figure 1:**
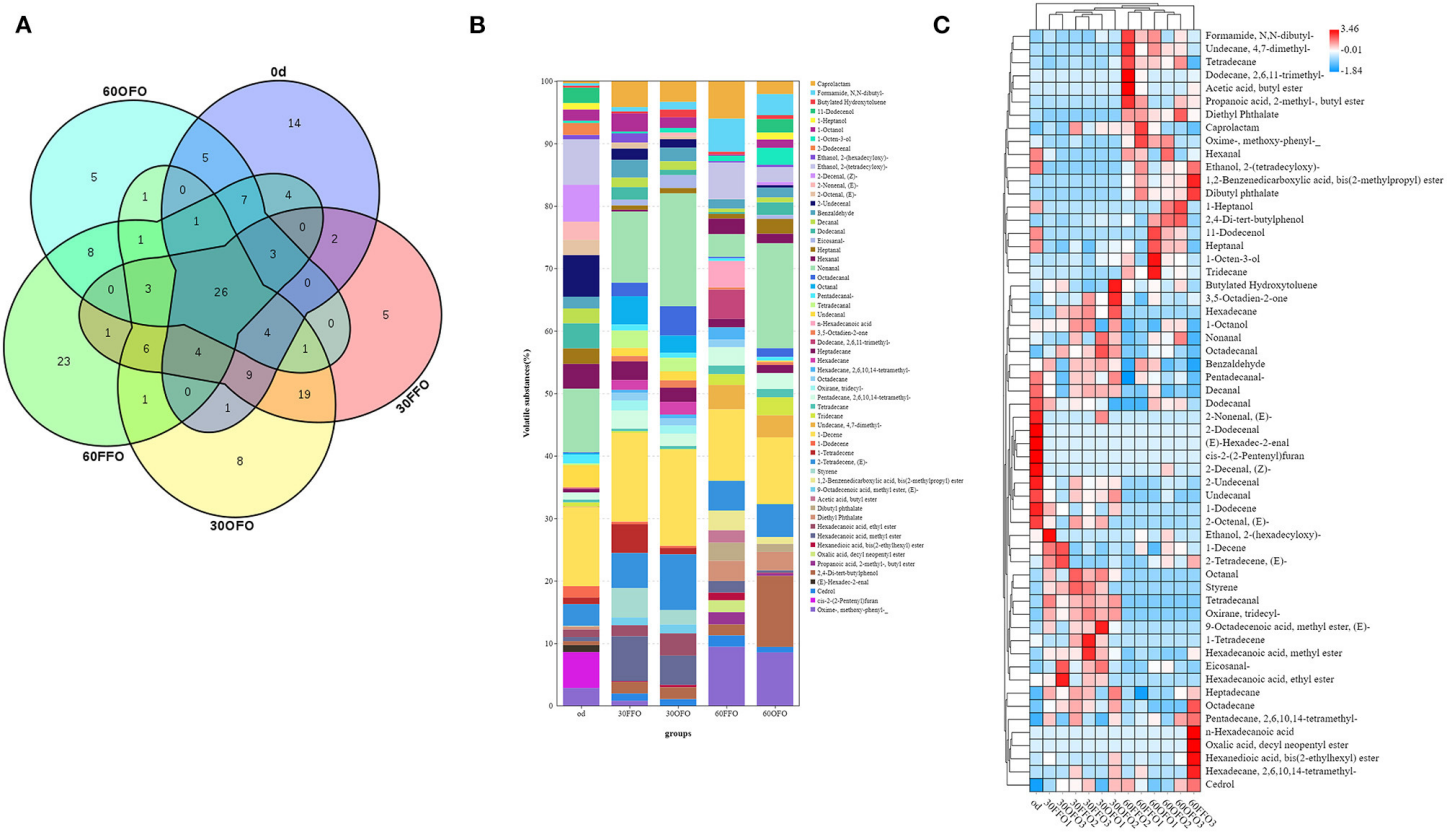
Muscle volatile compounds species of grouper fed different diets.

A variety of 162 volatile compounds were identified in grouper muscle (Figure 1A), including 80 species identified at the initial stage, 83 species at 30 days and 88 species at 60 days of FFO group; 85 species at 30 days, 57 species at 60 days of OFO group, and 26 of which are volatile compounds common to all groups. The abundance of volatile compounds in grouper muscle decreased significantly after 60 days of ingesting OFO feeds, with 31 species reduced compared to the FFO group. These 162 volatile compounds are mainly composed of amines, benzenes, alcohols, aldehydes, acids, ketones, alkanes, alkenes, esters, aromatic, etc. (Table 6). The content of alcohols and aldehydes in grouper muscle decreased significantly after ingestion of FFO feeds, while esters increased significantly with the duration of ingestion (*P* < 0.05). In comparison, alcohols in grouper muscle fed on OFO diet decreased first and then increased significantly (*P* < 0.05) with prolonged feeding time, reaching a level that had no significant difference from the initial value. The aromatics content, by contrast, increased significantly with duration of OFO feed ingestion (*P* < 0.05). There were no significant differences of volatile compounds in grouper muscle after 30 days fed with different FO (*P* > 0.05). After 60 days of ingestion, aldehydes, and aromatics were significantly enhanced in the OFO group, and esters were significantly reduced compared with the FFO group (*P* > 0.05).

**Table 6:** Muscle volatile compounds of grouper fed different diets %.

**Volatiles**	**0 day**	**30 FFO**	**30 OFO**	**60 FFO**	**60 OFO**
Amines	0.67, 0.00	4.21, 1.59	4.12, 1.43	9.70, 3.65	4.94, 2.02
Benzenes	0.29, 0.00	0.66, 0.14	1.37, 0.51	1.04, 0.29	0.62, 0.11
Alcohols	16.90, 0.00^Bc^	4.61, 0.94^A^	2.93, 1.3^a^	6.55, 1.24^A^	10.59, 0.78^b^
Aldehydes	45.37, 0.00^C^	32.09, 4.90^B^	36.91, 10.34	9.56, 1.73^A**^	29.00, 2.13**
Acids	0.13, 0.00	0.00, 0.00	0.32, 0.32	6.95, 5.58	0.83, 0.31
Ketones	1.54, 0.00	2.91, 0.66	3.10, 1.32	0.97, 0.50	1.24, 0.39
Alkanes	4.76, 0.00	11.07, 1.86	10.16, 2.93	20.96, 8.28	12.88, 3.01
Alkenes	17.15, 0.00	27.83, 8.13	26.28, 12.72	14.94, 3.35	14.67, 3.95
Esters	2.95, 0.00^A^	12.56, 4.72^AB^	11.56, 3.31	18.53, 3.68^B**^	6.00, 1.62**
Aromatics	0.57, 0.00^a^	1.60, 0.12	1.64, 0.49^a^	1.45, 0.89**	10.40, 1.16^b**^
Other	9.66, 0.00	2.45, 0.56	1.62, 0.59	9.36, 4.38	8.82, 3.26

The results are presented as the means ± SEM (n = 3). Different superscript capital letters in the same row indicate significant differences in the FFO group at different time point, and different lowercase letters indicate significant differences in the OFO group at different time point. ^*^Indicates significant differences of two treatments at 30 days, ^**^Indicates significant differences of two treatments at 60 days (P < 0.05).

In addition, we performed stacked and heat map modeling of volatile compounds with an average of over 1% for all groups (Figures 1B,C). As observed in Figure 1B, grouper muscle volatile compounds were mainly enriched in Nonanal and 1-Decene, followed by 2-Tetradecene, (E)-, and that the various volatile compounds differed with the type of feed consumed and the time of ingestion. Compared to the initial value of muscle in grouper, Caprolactam, Formamide, *N, N*-dibutyl-, Octadecanal, Octanal, n-Hexadecanoic acid, Dodecane, 2,6,11-trimethyl-, Undecane, 4,7-dimethyl-, 2,4-Di-tert-butylphenol, Oxime-, methoxy-phenyl-, etc. were significantly enriched at 30 or 60 days after feeding on the diets. While Ethanol, 2-(tetradecyloxy)-, 2-Decenal, (Z)-, 2-Undecenal, Dodecanal, Hexanal, Undecanal, cis-2-(2-Pentenyl) furan were mainly enriched in grouper muscle at the beginning of the experiment, and their contents gradually decreased or disappeared as the fish grew. Muscle volatile compounds of grouper were similar in the FFO and OFO groups at 30 days. Nevertheless, Caprolactam, Ethanol, 2-(tetradecyloxy)-, n-Hexadecanoic acid, Dodecane, 2,6,11-trimethyl-, Undecane, 4,7-dimethyl- Hexadecanoic acid, methyl ester content decreased sharply compared to the FFO group after 60 days of ingestion, while Non-anal and 2,4-Di-tert-butylphenol were significantly enriched. The clustering of volatile compounds in grouper muscle with ingestion time and type of feed consumed can be observed after grouping hierarchical clustering. The volatile compounds detected in grouper muscle could be clearly and visually observed after clustering with higher content of volatile substances in each treatment group, and the clusters formed multiple clusters, indicating that there were differences in the volatile compounds detected in different treatments.

### Muscle Relative MRNA Expression

**Figure 2:**
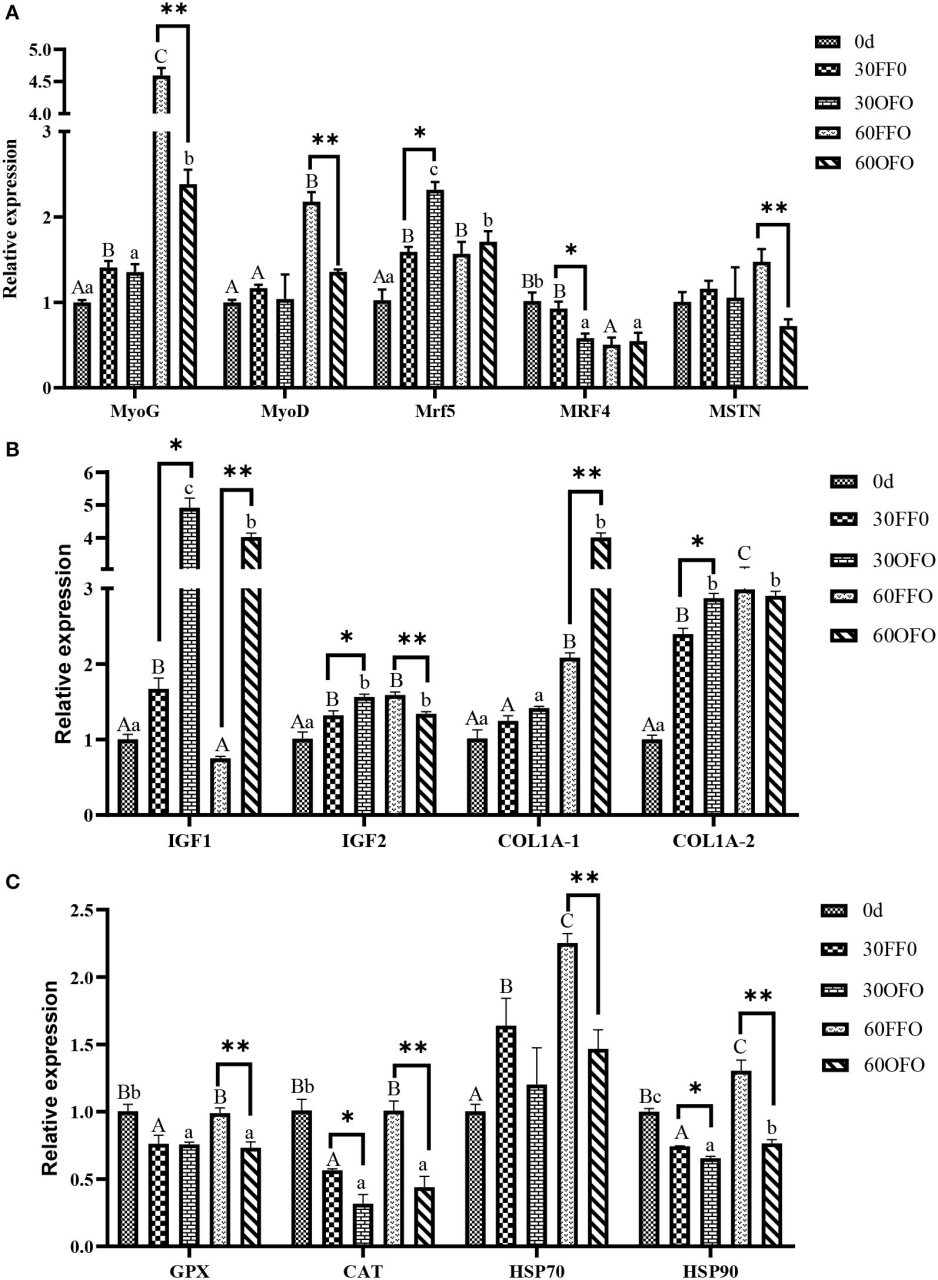
Relative expression of muscle growth factors **(A,B)** and antioxidant-related genes **(C)** mRNA of grouper fed different diets. *MyoG*, myogenin; *MyoD*, myogenic differentiation; *Myf 5*, myofactar5; *MRF4*, myogenic regulatory factor 4; *MSTN2*, myostatin type 2; *IGFl*, insulin-like growth factor 1; *IGF2*, insulin-like growth factor 2; *COL1A1*, collagen type I alpha1; *COL1A2*, collagen type I alpha2; *CAT*, catalase; *GPX*, glutathione peroxidase; *Hsp70*, heat shock protein 70; *Hsp90*, heat shock protein 90. Different superscript capital letters in the same row indicate significant differences in the FFO group at different time point, and different lowercase letters indicate significant differences in the OFO group at different time point. *Indicates significant differences of two treatments at 30 days, **indicates significant differences of two treatments at 60 days (*P* < 0.05).

The relative mRNA expressions of muscle growth factor in grouper were presented as in Figures 2A,B, myogenin (*MyoG*), myogenic differentiation (*MyoD*), insulin-like growth factor 2 (*IGF2*), collagen type I alpha1 (*COL1A-1*), and collagen type I alpha2 (*COL1A-2*) mRNA expression was significantly up-regulated and myogenic regulatory factor 4 (*MRF4*) mRNA expression was significantly down-regulated with prolonged ingestion of FFO diet (*P* < 0.05). Simultaneously, muscle *MyoG*, myofactar5 (*Mrf5*), insulin-like growth factor 1 (*IGF1*), *IGF2, COL1A-1*, and *COL1-2* mRNA expressions were significantly upregulated and *MRF4* mRNA expression was significantly down-regulated after ingestion of OFO diet (*P* < 0.05). Ingestion of OFO diet significantly upregulated *Myf5, IGF1, IGF2*, and *COL1A-2* mRNA expression and significantly decreased *MRF4* mRNA expression compared to the FFO group at 30 days (*P* < 0.05). However, ingestion of OFO diet significantly decreased *MyoG, MyoD*, myostatin type 2 (*MSTN 2*), *IGF2* mRNA expression and upregulated *IGF1* and *COL1A-1* mRNA expression compared to the FFO group at 60 days (*P* < 0.05).

The relative mRNA expressions of muscle antioxidant-related genes were illustrated in Figure 2C, consumption of OFO diet significantly decreased the mRNA expression of glutathione peroxidase (*GPX*), catalase (*CAT*), and heat shock protein 90 (*Hsp90*) in grouper muscles compared with those at the beginning of experiment (*P* < 0.05). Although the mRNA expression of *GPX, CAT*, and *Hsp90* in grouper muscle was significantly reduced by FFO ingestion at 30 days, it was significantly up-regulated and higher than the initial value at 60 days (*P* < 0.05). Furthermore, muscle heat shock protein 70 (*Hsp70*) mRNA expression in FFO group was significantly up-regulated with prolonged time (*P* < 0.05). Compared with the FFO group, ingestion of OFO diet for 30 days significantly decreased the mRNA expression of *CAT* and *Hsp90* and ingestion of OFO diet for 60 days significantly decreased the mRNA expression of *GPX, CAT, Hsp70*, and *Hsp90* of grouper muscle (*P* < 0.05).

## Discussion

As the most important part of fish for human consumption, muscle nutritional composition is particularly important. In present investigation, ingestion time and type of FO did not affect grouper muscle crude lipid and crude protein. However, Yu et al. (13) showed that ingestion of OFO significantly decreased muscle crude lipid and crude protein content in Amur sturgeon (*A. schrenckii*), this is probably due to differences in fatty acid utilization by different fish or different experimental periods, etc. Consumption of OFO has been shown to cause elevated MDA levels in various tissues of fish (14). The MDA level in the muscle of OFO group was higher than that of FFO group in the current study, which was consistent with previous study (15). Although there was no significant difference about the other indicators between OFO group and FFO group, OFO significantly increased the TC, and TG content of grouper muscle compared with the initial value, all of which reflected the toxic impact of OFO. With the growth of grouper, TG were accumulated significantly in the muscle after OFO ingestion, which also implied a downward tendency of muscle quality of fish due to lipid deposition caused by OFO (16).

The main indispensable amino acids in grouper muscle are lysine, leucine and arginine, and the main non-essential amino acids are aspartic acid and glutamic acid, in a recent report published from our team, these amino acid levels were similar to the results of the present study (17). Indispensable amino acid/total amino acid (IAA/TAA) ratio is an important reference indicator for evaluating the nutritional value of aquatic protein, and it is generally considered that the ideal IAA/TAA ratio for high quality protein is about 0.4 (18), the ratio of IAA/TAA in grouper muscle in the present study was ~0.5, indicating that grouper muscle is a high-quality protein source. Methionine, phenylalanine, total indispensable amino acid, aspartic acid, serine, glutamic acid, total dispensable amino acid, and total amino acids of muscle in FFO group first decreased significantly and then increased to no significant difference from the initial, suggesting a self-regulatory ability of fish muscles. Proline content increased significantly with ingestion time, regardless of the type of feed consumed. In biological organisms, proline is not only an ideal osmoregulatory substance, but also serves as a protective substance for membranes and enzymes and a free radical scavenger, and provides the material basis for growth promotion, normal metabolism and life maintenance, therefore, proline may play an important role as a functional amino acid in regulating muscle growth and metabolism in grouper. Phenylalanine, as a sweet amino acid recognized by the Joint Committee of Experts of the World Health Organization (WHO) and the Food and Agriculture Organization (FAO), was significantly higher in the OFO group than in the FFO group after 60 days of ingestion, which to a certain extent indicates that the OFO changed the amino acid profile of muscle.

Fatty acid composition of fish tissues was influenced by fatty acid composition of ingested diets (19), the significant decrease in muscle SAFA and MUFA and the significant accumulation of PUFA with prolonged FFO ingestion in this study, further illustrates the importance of FFO as a recognized source of high-quality commercial lipids (20). The PUFA of OFO group was significantly lower than that of FFO group at 60 d, which may be attributed to the fact that part of the LC-PUFA in FO was oxidized to alcohols, aldehydes, acids, ketones, etc. (21), reduced the content of PUFA in FO and thus affected the accumulation of PUFA (especially EPA and DHA) in muscle, this is consistent with the results that OFO adversely affects the fillet quality of Amur sturgeon (*A. schrenckii*) (13).

In addition to muscle nutritional quality, sensory characteristics also determine the degree of consumer consumption of aquatic products, of which flavor is one of the important factors influencing the determination of sensory characteristics (22). The diversity of muscle volatile compounds decreased from 88 to 57 at 60 days on OFO diet, which reflects the destructive effect of OFO on muscle flavor diversity to a certain extent. In present study, aldehydes, esters, and aromatics were the main differential volatile compounds identified. Aldehydes with low threshold can be formed rapidly in lipid oxidation, so they contribute greatly to the formation of flavor substances in aquatic animals (23). Studies demonstrate that aldehydes are related to the grassy taste (23), fishy taste, and greasy taste of aquatic products (24) and account for a large proportion of the volatile substances. Esters compounds are formed by esterification of carboxylic acids and alcohols, and esters mostly give a fruity or floral flavor to foods (25). The aldehydes enriched in the present project were mainly non-anal, which as a saturated straight chain aldehyde mainly produced fishy and irritating odor and gave an unpleasant smell (24), and its content decreased significantly after ingestion of FFO diet and increased significantly after ingestion of OFO diets, and it was significantly higher in OFO group than that in FFO group at 60 days. While the ester content accumulated significantly in FFO group and was significantly higher than that in OFO group at 60 days. These differential substances may be derived from the interaction between the Maillard reaction (26) and lipid oxidation. In addition, compared with the FFO group, the level of Aromatics such as 2,4-Di-tert-butylphenol was significantly increased by the consumption of OFO diet. Some studies have suggested that the substances may be transferred to the fish by environmental pollutants and may produce negative effects on fish muscle (27), but in the present study, all conditions remained the same except for the difference in feed consumption, which, in our opinion, on the one hand, it may be the result of the toxic effect of MDA and peroxide in OFO; on the other hand, may be due to the low utilization of nutrients, especially lipid, caused by the oxidation of lipids in the body caused by the consumption of OFO, resulting in the excretion of nutrients that further polluted the aquaculture environment. Of course, only our speculation, the specific reasons still need further verification. These indicated that FO itself was a good source of lipids to enhance flavor, but being oxidized would make a considerable negative consequence on the flavor and quality of aquatic products, and even affect the sales of aquatic products.

A variety of molecular mechanisms regulate the physiological processes, degeneration, and quality of muscle growth (28). With specific aspects of muscle growth and catabolism dependent on nutritional condition, the expression of genes related to myogenic signaling pathways was frequently analyzed to determine the molecular response to various diets (29). *MyoG, MyoD, Myf5, MRF4, MSTN 2, IGF1, IGF2, COL1A-1*, and *COL1A-2* are all myogenic regulatory factors (MRFs) growth-related factors, which are internal factors that control specification, activation, and differentiation of myogenic cells (30). *MyoG* plays an essential part in myoblast differentiation, *MyoD* and *Myf5* are primarily myogenic determinants (31). The expressions of *MyoG, MyoD*, and *Myf5* were up-regulated after ingesting two diets, the results indicated that OFO has no adverse effect on muscle differentiation and formation in grouper. However, the expression of *MyoG* and *MyoD* in the muscle of grouper consuming OFO diet for 60 days was significantly lower than that of the FFO group, this also illustrated the negative effect of OFO on grouper muscle differentiation. It also confirms that *MyoG* and *MyoD* levels always seem to correlate with nutritional status (32). Also, muscle is regulated by MSTN, which normally inhibits the growth of skeletal muscle (33). *IGF1* is a key positive regulatory growth factor with abundant receptors in fish muscle that promotes cell activation, proliferation and differentiation (34); whereas, *IGF2* is a negative regulator (35). In turn, *COL1A-1* and *COL1A-2* regulate the diameter and stiffness of muscle fibers (36). Although muscle *IGF1, COL1A1*, and *COL1A2* were significantly upregulated after feed intake, *IGF1, COL1A1* were significantly higher in OFO group than those in FFO group, and correspondingly, the expression of *MSTN* and *IGF2* was downregulated. This might be due to the increase of MDA and TG content in the muscles of grouper feeding on OFO feed, and the deposition of lipid and harmful substances play a certain stimulating effect on the growth of muscles, showing negative feedback regulation. We speculate that the ingestion of OFO may lead to the inhibition of myotubular value-added and the reduction of fusion number in grouper muscles, and inhibit the proliferation and differentiation of myoblasts, but the by-products produced may play a certain negative feedback regulation on the growth of muscles, which may also be the reason why the ingestion of OFO fish can grow normally but with poor quality, but these speculations need to be further verified.

*GPX, CAT, Hsp70*, and *Hsp90* are all antioxidant-related factors in the body. CAT could make a dynamic balance between the production and elimination of free radicals and reduce oxidative damage (37). GPX catalyzes the breakdown of peroxides, thereby protecting the structural and functional integrity of cell membranes (38). *Hsp* factors can modulate stress tolerance by preventing protein denaturation, refolding damaged proteins, or ensuring irreversible degradation of damaged proteins (39). OFO intake down-regulated the expression of muscle *GPX, CAT, Hsp70*, and *Hsp90* compared with the initial values or those of FFO group at the same time points. The results implied that OFO caused damage to the antioxidant system of grouper muscles, this corresponded to the above results of elevated MDA and TC levels in grouper muscle caused by OFO.

## Conclusion

To overview, ingestion time and diet lipid type both could affect the muscle quality of grouper, while the effect of diet lipid type is the main factor. OFO may increase the deposition of harmful substances and lipid in muscle through oxidative damage, thus affecting nutrient metabolism and reducing muscle nutritional quality, while oxidative decay of PUFA in FO is an important factor in muscle flavor deterioration, and the mechanism needs further investigation. The project is expected to provide reference data for the feed industrial use of FO.

## Data Availability Statement

The original contributions presented in the study are included in the article, further inquiries can be directed to the corresponding author/s.

## Ethics Statement

The animal study was reviewed and approved by the Ethics Review Board of the Institutional Animal Care and Use Committee at Guangdong Ocean University in Zhanjiang, China.

## Author Contributions

XY: data analysis and manuscript writing. ZL: experimental implementation. XD: experimental design and manuscript revision. BT: experimental design. SP, TL, SL, WH, and XS: sample analysis. YY: data management. All authors contributed to the article and approved the submitted version.

## Funding

This study was supported financially by the National Natural Science Foundation of China (NSFC31972808), the General Program of Natural Science Foundation of Guangdong Province (2021A1515011165), Department of Education of Guangdong Province (2021ZDZX4005), Postgraduate Education Innovation Project of Guangdong Ocean University (202139), Science and Technology Bureau of Zhanjiang (Grant No. 2020A03010 and 2020A05003), supported by China Agriculture Research System of MOF and MARA (CARS-47), and Research and Demonstration of Precision Functional Compound Feed Technology of Major Cultured Fishes and Shrimps in South China (2021B0202050002).

## Conflict of Interest

The authors declare that the research was conducted in the absence of any commercial or financial relationships that could be construed as a potential conflict of interest.

## Publisher's Note

All claims expressed in this article are solely those of the authors and do not necessarily represent those of their affiliated organizations, or those of the publisher, the editors and the reviewers. Any product that may be evaluated in this article, or claim that may be made by its manufacturer, is not guaranteed or endorsed by the publisher.
