# Supplementary figures and images for: Degradation of Muscle Quality in Hybrid Grouper (♀ Epinephelus fuscoguttatus × ♂ Epinephelus lanceolatu) Due to Oxidative Damage Caused by Ingestion of Oxidized Fish Oil
Source: Front Nutr. 2022 Feb 15;9:840535. doi: 10.3389/fnut.2022.840535 (PMC8886721; doi:10.3389/fnut.2022.840535)

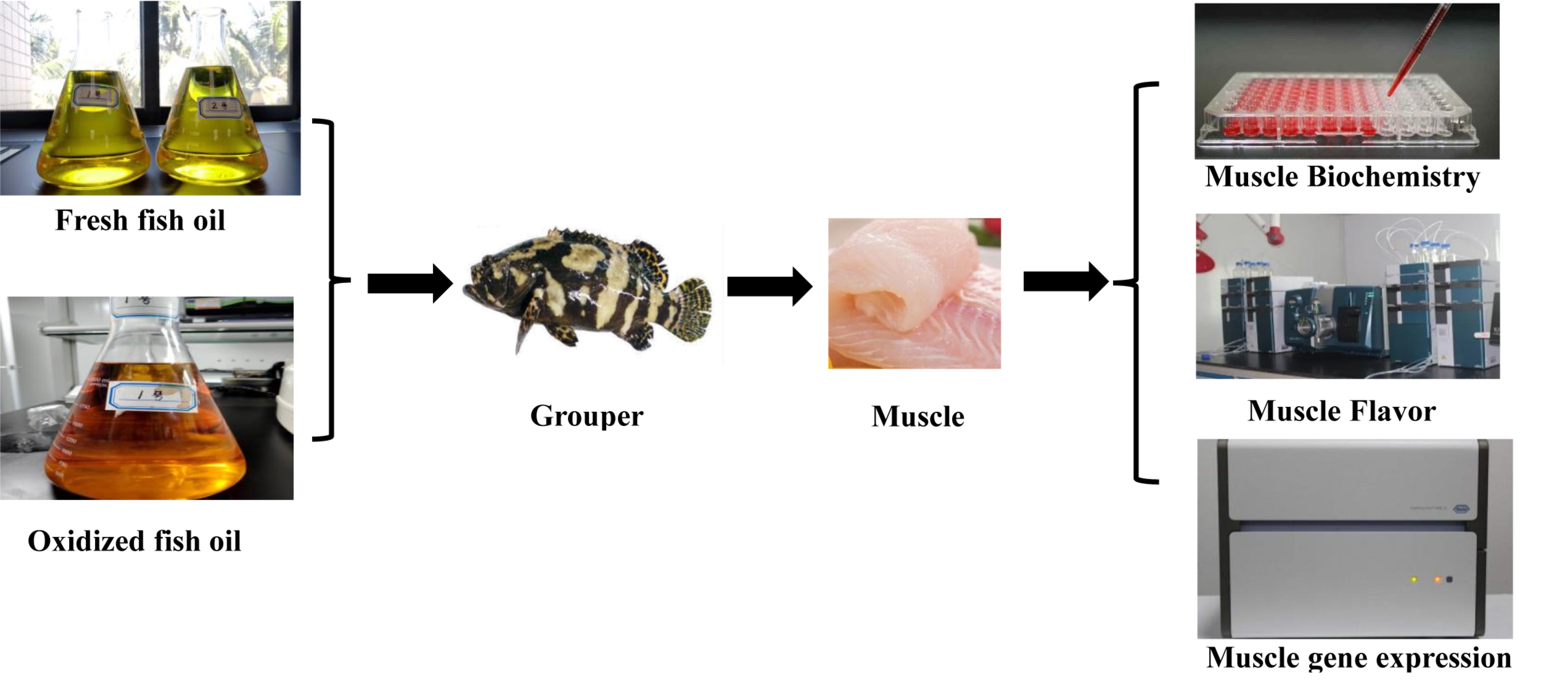

Supplement: Supplementary file 1 [file Image_1.TIF]
